# Egg Size Effects across Multiple Life-History Stages in the Marine Annelid *Hydroides diramphus*

**DOI:** 10.1371/journal.pone.0102253

**Published:** 2014-07-18

**Authors:** Richard M. Allen, Dustin Marshall

**Affiliations:** School of Biological Sciences, University of Queensland, St Lucia, Queensland, Australia; Gettysburg College, United States of America

## Abstract

The optimal balance of reproductive effort between offspring size and number depends on the fitness of offspring size in a particular environment. The variable environments offspring experience, both among and within life-history stages, are likely to alter the offspring size/fitness relationship and favor different offspring sizes. Hence, the many environments experienced throughout complex life-histories present mothers with a significant challenge to optimally allocate their reproductive effort. In a marine annelid, we tested the relationship between egg size and performance across multiple life-history stages, including: fertilization, larval development, and post-metamorphosis survival and size in the field. We found evidence of conflicting effects of egg size on performance: larger eggs had higher fertilization under sperm-limited conditions, were slightly faster to develop pre-feeding, and were larger post-metamorphosis; however, smaller eggs had higher fertilization when sperm was abundant, and faster planktonic development; and egg size did not affect post-metamorphic survival. The results indicate that egg size effects are conflicting in *H. diramphus* depending on the environments within and among life-history stages. We suggest that offspring size in this species may be a compromise between the overall costs and benefits of egg sizes in each stage and that performance in any one stage is not maximized.

## Introduction

Understanding the evolutionary ecology of offspring size is a central component of life-history theory. Larger offspring typically have higher fitness (e.g., in plants [1], [2], invertebrates [3], [4], and vertebrates [5], [6], [7]), but because the resources available for reproduction are limited, mothers must maximize their own fitness by balancing the benefits of producing larger, fitter offspring with the costs of decreased fecundity [8], [9]. A-single, optimal offspring size however is unlikely for any given species, because the strength and direction of the offspring size-fitness relationship is mediated by the environmental conditions experienced by the offspring [9], [10], [11], [12], [13], [14], [15], [16]. In some environments, offspring size effects on performance are positive and strong, and so selection favors mothers that increase their own fitness by producing larger offspring [14]. In other environments, selection can be positive but weak, absent, or negative; in these environments, selection will favor mothers that produce smaller, but more numerous offspring [17], [18], [19]. Such environmentally-driven variation in the relationship between offspring size and offspring fitness presents mothers with a significant challenge to optimally allocate their reproductive effort, and can lead to significant variation in offspring size within a population [20].

The challenges of optimally provisioning offspring are exacerbated in species with complex life-histories. Individuals can experience very different environments as propagules, larvae, and adults; and, the different environments encountered by offspring within each life-history stage may favor a different offspring size [21] (but see [22], [23], [24]; and [25] for other traits). More generally, complex life-cycles can allow organisms to adapt independently to the ecologically distinct environments of each stage [26], but offspring size is not a reversible trait: once an offspring is independent of its mother, the mother cannot reclaim any unused resources, nor supplement any deficiencies. For example, there is conflicting selection on seed size in oak seedlings: larger seeds have higher fitness during early ontogeny (such as germination rate, and seedling growth and survival), but suffer higher post-dispersal mortality due to size-selective predation [23]. Similar examples are found in salmon, frogs and marine invertebrates [18], [21], [24]. The optimal offspring size for a given population will therefore be a compromise between offspring size effects across the entire life-history, but may be constrained by highly sensitive stages or those under the most intense selection [24], [27].

Critical to the argument that conflicting pressures across multiple life-history stages can shape an optimal offspring size is the assumption that initial differences in offspring size can persist to affect later life-history stages [28]. In species with a short and non-feeding larval stage, we see evidence that offspring size and maternal effects can persist into adult life, and evidence of variable or conflicting effects on offspring size [23], [28], [29]. However, offspring size and other maternal effects can dissipate over the course of development and contribute little to adult fitness (e.g., [30], [31], [32]). Failure to detect these effects in later stages is often attributed to the environment contributing more to subsequent adult phenotypes than maternal effects [33], [34]. This appears to be particularly true for species with feeding developmental stages, because variation in the nutritional environment experienced by the offspring can correlate with adult nutrition and phenotypes, and overwhelm egg size effects [35]. Furthermore, small offspring may supplement deficiencies in maternal provisioning by prolonging development or accelerating growth/feeding rates to acquire more exogenous resources and achieve comparable size at maturity to larger offspring ([3], [17], [36], [37] [38] [39]. Although a feeding larval stage is common in most animal taxa (e.g., most insects, amphibians, fish, and marine invertebrates), few studies have determined the persistence of offspring size effects in species with this type of life-history. In part, this may reflect logistical difficulties in tracking individuals with complex life-cycles across different stages, so studies that examine conflicting selection on offspring size are exceedingly rare in species with feeding larvae.

Here, we examine the effects of natural variation in egg size on performance across multiple life-history stages in *Hydroides diramphus. Hydroides diramphus* has a complex life-cycle consisting of egg, embryonic, larval, and post-metamorphic stages. Experimentally, we test to see if egg size effects can persist across multiple life-history stages, and in doing so, we identify the potential range of life-history stages that may be influenced egg size-performance relationships. Additionally, we examine whether egg size influences proxies of offspring performance among each stage of these stages to describe when different offspring sizes should be favoured.

## Materials and Methods

### General Methods

#### Study species and site

*Hydroides diramphus* is a polychaete tubeworm found in benthic marine assemblages and is a non-native species in Australia. All collections and field experiments were conducted between May and September 2008 from the floating docks at the Scarborough Marina, Redcliffe, Queensland, Australia (27° 10' 45" S, 153° 06' 18" E). The floating docks are privately owned and managed by the Moreton Bay Boat Club, and permission was granted by club management for the collection of specimens and complete experiments. Worms were collected and transported to the laboratory in thermally insulated tanks for spawning and larval culture.

In *H. diramphus* the sexes are separate, and fertilization occurs externally. Generally, the fertilization environment is highly variable in natural populations and is strongly influenced by proximity to mates [40]. Many marine invertebrates spawn synchronously, and at high densities fertilization can be as high as 100% in some species [41], [42], [43]. In *H. diramphus*, individuals can touch or be millimeters apart at high densities, but also be meters apart at low population densities (Allen personal observations). Therefore, natural fertilization in *H. diramphus* likely occurs across a range of sperm concentrations from very dense when female and male tubes overlap to approaching zero where mates are far apart. Importantly, the fertilization environment can be a selective pressure on egg size in marine invertebrates [27]. Because larger eggs present a larger target to sperm, under low-sperm concentrations, larger eggs have higher fertilization success; but at high-sperm concentrations, large eggs can suffer polyspermy: a fatal condition that results from eggs being fertilized by multiple sperm [27], [46].

Once fertilized, *H. diramphus* embryos develop for 12–14 hrs and hatch into obligate feeding (planktotrophic) larvae. Under natural conditions, mortality is generally high during embryonic and larval development, and only a small fraction of the number of offspring produced by mothers typically recruit back to adult populations [45]. In other taxa, size-dependent mortality during development has been documented (e.g., [46], [47]), but in marine invertebrates, size dependent mortality has not been explored. More generally, the specific sources of mortality remain largely unknown in marine invertebrates, but larval mortality is usually reported as an instantaneous rate function of the time spent in the plankton [45], [48]. Importantly, the relationship between offspring size and development time has strong theoretical foundations derived from allometric and kinetic principles (e.g., [49]) and variable developmental relationships among marine taxa have been demonstrated by empirical studies [4]. Hence, the offspring size-development time relationship is a good proxy for probability of planktonic survival in marine invertebrate larvae [50].

Once *H. diramphus* larvae acquire enough resources during planktotrophic development, larvae become competent to settle and metamorphose into the adult environment. In the post-metamorphic environment, egg size may also be under selection, because other studies on marine invertebrates with different life-histories have shown that offspring size (as eggs or larvae) can influence growth, competitive ability, survival and reproductive output [4], [51], [52].

#### Obtaining gametes and larval culture techniques

In the laboratory, gametes were obtained using a strip spawning technique [53]. Worms were gently removed from tubes and placed in 30 mm diameter petri dishes with <5 ml of sterilized seawater (seawater microwaved at 1000 watts for 3 min past boiling and allowed to return to ambient temperature). Reproductively mature individuals released gametes immediately. Fertilization techniques varied with experiments (see below for details), but generally, eggs were placed in a beaker with a few drops of fresh sperm obtained from males immediately prior to fertilization. To ensure maximum fertilization and avoid sperm concentration selecting for particular egg sizes, the drops of sperm were added in a stepwise manner at 10–15 min intervals so that concentration of sperm gradually increased, but also allowed time for the polyspermic block to take place. At 22°C, trochophore larvae hatch from developing embryos at approximately 12–14 hours. Trochophore larvae were reared at concentrations of 50 larvae ml^−1^, and fed *Isocrysis galbana* (Tahitian strain) in standing cultures at concentrations of 100,000 cells ml^−1^. Fresh, sterilized seawater and phytoplankton were replaced every second day. Competency to settle and metamorphose was reached between 6 and 18 days after fertilization, and was determined by morphological examination of larvae [54].

#### Measurements

Egg size was used to estimate maternal investment. We recognize that egg size does not correlate perfectly with maternal investment in all species [55]; however, among annelids at least, egg size does correlate with the energy content of eggs [56]. Furthermore, three additional selective mechanisms may operate on egg size independently of energy content: 1) mothers face a physical constraint of brood-space [4]; 2) fertilization success is determined by physical egg size (outlined above, [27], [57]; and 3) egg size is a trait typically related with development mode and other life-history traits under selection in marine invertebrates and other taxa [3], [4], [6].

To measure eggs and larvae, samples were fixed in 5 ml seawater with a few drops of 12% formalin. Eggs and larvae were photographed with a compound microscope and camera (PixeLINK Capture SE Ver. 1.0). We measured the two-dimensional cross-sectional area of eggs and pre-feeding trocophore larvae (lateral view). Competent larvae were also measured as the cross-sectional area along a longitudinal orientation. The calcareous tubes that *Hydroides* secrete and inhabit are enlarged as juveniles develop to accommodate growth. Hence, tube length is a good indicator of worm size and growth in *Hydroides* species [58], [59]. The relationship between tube length and worm size is strongest after 9 days in similar *Hydroides* species [58], so at 10 days in the field, worms were returned to the laboratory and tube length was measured under a dissecting microscope. Maternal worms were measured out of their tubes as length from the most anterior part of the collar to the most posterior tip of the abdomen. All traits on digital images were measured with Image-Pro express version 5.1.

#### A natural source of egg size variation: the relationship between egg size and maternal size

The first goal of the study was to describe the existing amount of variation in egg size for the *H. diramphus*. There are many potential sources of variation in offspring size that are important to many aspects of life-history theory and ecological relevance, and are correlated with maternal traits [60]. One of the more common maternal effects on offspring size is the relationship between maternal size and offspring size [4], [60]. To describe the standing variation in egg size, and investigate whether maternal size is a natural source of egg size variation, we examined the relationship between maternal size and egg size. We spawned and measured 26 females, and sampled 25 eggs from each mother. The average egg size per mother was used as the unit of replication to estimate the relationship between maternal size and egg size.

### Egg size effects during early development

The aim of the following experiment was to investigate how egg size and the fertilization environment mediate the maternal-offspring size relationship and affects fertilization success. A pilot study revealed the relationship between sperm concentration and fertilization success conformed to the fertilization curve found in other marine invertebrates (e.g., urchins, [57]: and polychaetes, [61]). We found fertilization success peaked at ∼23×10^6^ sperm ml^−1^ with 88% of eggs fertilized, and at a sperm concentration of ∼23 sperm ml^−1^, fertilization success was 3.5% (Allen RM unpublished data). We used these two sperm concentrations to represent the extremes in selection pressure due to fertilization success. Sperm concentrations were determined by three replicate counts of sperm samples using a haemocytometer. In polychaetes, unfertilized eggs may cleave and polyspermic embryos may also continue to develop, but neither unfertilized nor polyspermic embryos develop past hatching. Hence, larval size of newly hatched larvae was the response variable of interest and a proxy for the size of eggs that were successfully fertilized (because larger eggs produce larger larvae; see *Results: Egg size effects during early development*). Twenty-three mothers were spawned, and the broods split into two separate petri dishes; then the fertilized the eggs from each mother at either the ‘high’ or ‘low’ sperm concentrations. At hatching, we measured the size of larvae from each mother and sperm concentration. Maternal size was also recorded as a proxy for egg size, and each mother was the unit of replication.

The aim of the next experiment was to investigate the effects of egg size on early development time, using time-lapse photography to measure early cleavage rates. We placed a sample of fertilized embryos in a 30 mm diameter petri dish under a dissector microscope. One image was taken every minute for 90 minutes. We followed the development of individually measured eggs from first cleavage (2-cell stage) to second cleavage (4-cell stage) to estimate early development time. We did not measure from time of fertilization to subsequent stages because we could not account for variation in exact moment each egg was fertilized, and we did not measure past the 4-cell stage because differentiating further stages was unreliable at the image resolution.

We then tested whether egg size affected the size of larvae. Twenty-six mothers were spawned and a sample of 25 eggs from each brood measured; then the remaining eggs fertilized in separate petri dishes (one dish per brood). As described in the *Obtaining gametes and larval culture techniques* section, we avoided selecting against particular egg size by fertilizing eggs in the stepwise manner described above. We allowed the embryos to develop to hatching, where we then fixed and measured a sample of 25 newly hatched larvae from each dish.

### Egg size effects during planktonic development

Measuring a single egg and following that individual through multiple life-history stages is logistically very difficult to replicate in large numbers in most free-spawning marine invertebrates, because of their small size, and sensitivity to laboratory culture. Hence, instead of using maternal and egg size as covariates, we grouped mothers into ‘large’ and ‘small’ size classes that would enable the manipulation of egg size at the level of culture jar to investigate maternal size and egg size effects during planktonic development. To achieve this, we spawned 60 mothers, and ranked them roughly from largest to smallest. We then omitted the six mothers closest to the median in order to reduce any overlap in egg sizes. The eggs from large and small mothers were pooled within each size treatment, then immediately split into experimental jars. By grouping mothers into size classes and removing intermediately sized mothers, we improved the reliability that the large maternal size class contained large eggs. Approximately, 75% of the variation in egg sizes among replicate jars at the beginning of the experiments that used this procedure (see *Results*).

The goal of the next experiment was to investigate the effect of maternally derived egg size variation on planktonic development time to settlement. Like most species with a planktotrophic larval stage, *H. diramphus* larvae must pass through an obligate feeding stage where sufficient resources are required to settle and metamorphose. As described above, eggs from 60 mothers were sorted into size classes and split into 20 experimental jars (10×600 ml beakers of each size class) containing 250 ml of sterilized seawater. We fertilized the eggs in each jar with sperm pooled from five males; again, using step-wise fertilization to avoid selecting for particular egg sizes (*Obtaining gametes and larval culture techniques*). The embryos developed to hatching, after which the water was changed, and the concentration of larvae adjusted to ∼50 larvae ml^−1^ across all jars. Phytoplankton was added to each jar (*Isocrysis* 100,000 cells ml^−1^) and the larvae were allowed to develop. To ensure all larvae remained in the culture during water changes, we placed a 25 µm cylindrical-filter into the culture vessel, and from inside the filter, we pippetted, then discarded, the waste water. The front of the filter was thoroughly rinsed to retain all larvae within the culture. Settlement was measured as the proportion of larvae that had settled every three days for fifteen days, at which time almost all larvae settled (see *Results*). In some circumstances, marine invertebrate larvae may have a prolonged planktonic period because suitable sites for sedentary adult life are rare, or food limitation slows larval development (e.g., [62], [63]). Both instances could affect the size of larvae at settlement (e.g., [62], [63]). Ample food was provided (at a concentration above what larvae can consume before food was replaced) and *H. diramphus* larvae readily settle on surfaces with a biofilm, so settlement represents a ‘best case’ scenario where larvae can settle and metamorphose at a time that suits them.

### Egg size effects on post-metamorphic performance

To examine the effect of egg size on performance after metamorphosis, we repeated the above procedures for spawning, fertilization, larval feeding and sorting of eggs into two size classes, but in this experiment eggs were split into 16 replicate jars (8 jars of ‘large’ and ‘small’ eggs each).

After 10 days, a high proportion of larvae were competent to settle, so the larvae from each experimental jar were transferred into corresponding petri dishes for settlement (60 mm diameter; 16 jars, 16 dishes). Dishes were pre-roughened and conditioned in natural seawater for at least three days prior to settlement, because many marine invertebrates prefer to settle on a roughened surface with a biofilm [64]. Once larvae were added, dishes were left for 48 hrs to allow larvae to settle and metamorphose. Once settled, ∼20 larvae per dish were marked by circling individuals with a graphite pencil. Any extra settlers or unsettled larvae were haphazardly culled and discarded. A 6 mm hole was drilled in the centre of each dish, and the dishes were transported to the field where they were fixed to a 500×500 mm backing panel and hung one meter below the floating docks. Dishes were left in the field for 10 days, after which dishes were returned to the lab, and scored for survival and tube-length was measured. All surviving worms were measured, and the average length of worms per dish was the unit of replication.

### Data analysis

All data were analyzed using Systat ver. 11 and checked for the assumptions of each statistical test. Where data sets were not normally distributed, they were transformed as indicated for each test below. Where no transformation is indicated, data did not require transformation. Data are available in supplementary File S1.

We analyzed the relationship between maternal body size and average egg size; maternal size and coefficient of variation in egg size; egg size and larval size; and egg size and early development time with linear regression. We also checked whether sorting eggs into size class based on maternal size were significantly different with a one-way ANOVA.

The effect of sperm concentration on the size of larvae that hatched was analyzed with ANCOVA where sperm concentration was the categorical variable and maternal size (as a proxy for egg size) was the covariate. Average larval size per dish was the response variable.

We analyzed the effect of larval size on planktonic development time with repeated measures ANOVA, where larval size was a fixed factor, and jar was the unit of replication. The proportion of larvae settled was the response variable, transformed by log_10_ to normalize the distribution. The Greenhouse-Geisser eigenvalue was low, and therefore the Greenhouse-Geisser adjusted *P*-value was reported as the test of significance because it is a more conservative test [65].

The effect of maternal size class on post-metamorphic size and survival was analyzed with a one-way ANOVA. The post-metamorphic size data distribution was skewed, and was square-root transformed for analysis. The proportional survival data were also not normally distributed, and were arc-sin square-root transformed for analysis.

## Results

### Relationships between maternal size and offspring size

**Figure 1:**
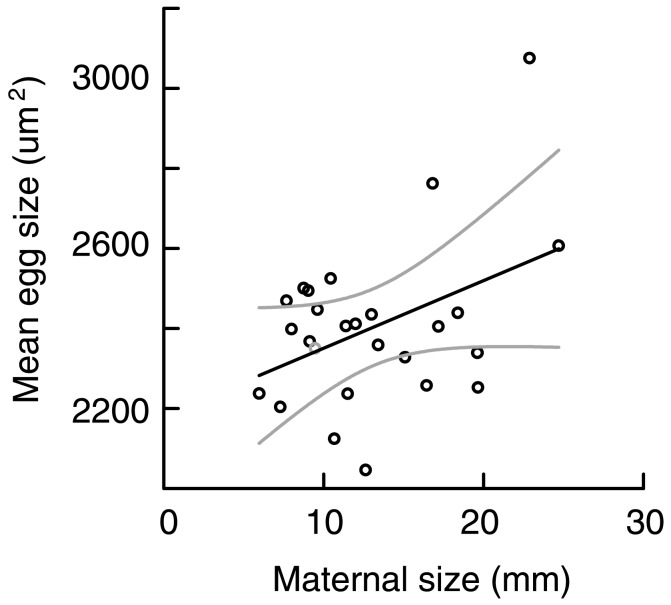
The relationship between maternal worm length and average egg area per mother (*P* = 0.034, R^2^ = 0.175) in *Hydroides diramphus*. Solid lines represent significant line of best fit; dotted lines represent 95% confidence intervals.

Mothers in the study were on average 13.1 mm in length (±5 mm SE; CV  = 0.382). Among mothers, the mean egg size was 2403.22 µm^2^ (measured as 2-D surface area; ±202.3 µm^2^ SE). The coefficient of variation in egg size was 0.084 among all mothers (based on average egg size per mother), and the average CV within each mother was 0.101. Maternal body length was positively correlated with average egg area (*F*_1, 24_ = 5.083, *P* = 0.034, R^2^ = 0.175). On average, doubling the length of mothers from 10 to 20 mm resulted in an approximately 11.4% increase in the area of the eggs produced by mothers (Figure 1).

### Egg size effects during early development

**Figure 2:**
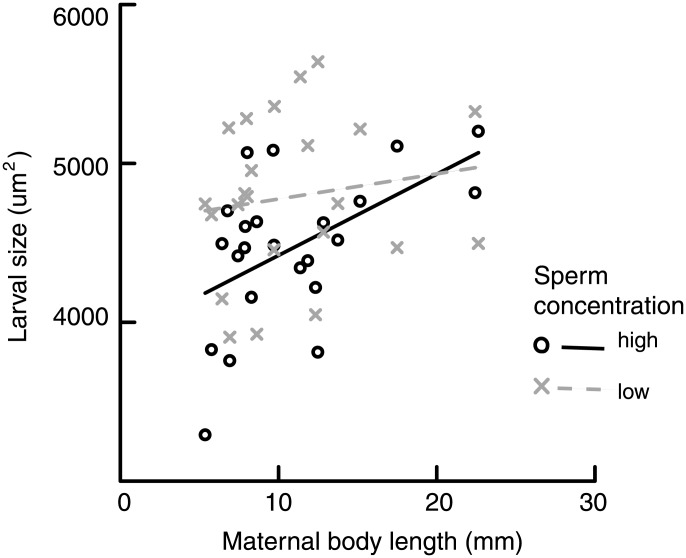
The effect of sperm concentration and maternal size on the size of larvae that complete development to hatching in *Hydroides diramphus*. Low and high sperm concentrations were ∼23 sperm ml^−1^ (× and dotted line) and ∼23×10^6^ sperm ml^−1^ (○ and solid line), respectively.

The sperm environment in which eggs were fertilized significantly affected the size of newly hatched larvae, where larvae from eggs fertilized under low-sperm conditions had an average cross-sectional area that was ∼10% larger than the larvae from eggs fertilized under high-sperm conditions (*F*_1, 42_ = 4.255, *P* = 0.045; Figure 2). Larval size was positively correlated with initial egg size (see below), therefore, the differences in larval size between treatments is likely to reflect different fertilization success due to egg size, where larger eggs are fertilized more frequently under low sperm concentrations. Maternal length was positively related to larval area, independent of sperm concentration (*F*_1, 42_ = 5.390, *P* = 0.025; Figure 2). There was no interaction between sperm concentration and maternal size (*F*_1, 42_ = 1.50, *P* = 0.227).

Once fertilized, larger eggs developed significantly faster than smaller eggs (*F*_1, 69_ = 5.252 *P* = 0.025): on average, eggs that were 50 µm in diameter took ∼8 min longer to develop from the 2-cell stage to the 4-cell stage, than eggs that were 60 µm in diameter. This effect of egg size on cleavage rates was weak: only 7% of the variation in development time could be explained by egg size (R^2^ = 0.071).

Larger eggs developed into larger larvae at hatching (*F*_1, 23_ = 9.665, *P* = 0.005, R^2^ = 0.296); although, the eggs from one replicate did not develop into larvae. The overall effect was significant, where on average, an increase in egg area of 25% resulted in a 4.7% increase in the cross-sectional area of the newly hatched trocophore larvae.

### Egg size effects during planktonic development

We checked that using maternal size class was an effective way of manipulating egg size variation, and found it was more reliable than maternal size as a covariate: eggs in the large size class were 15% larger in volume than eggs in the small size class (*F*_1,14_ = 42.73, *P*<0.001, R^2^ = 0.753).

**Figure 3:**
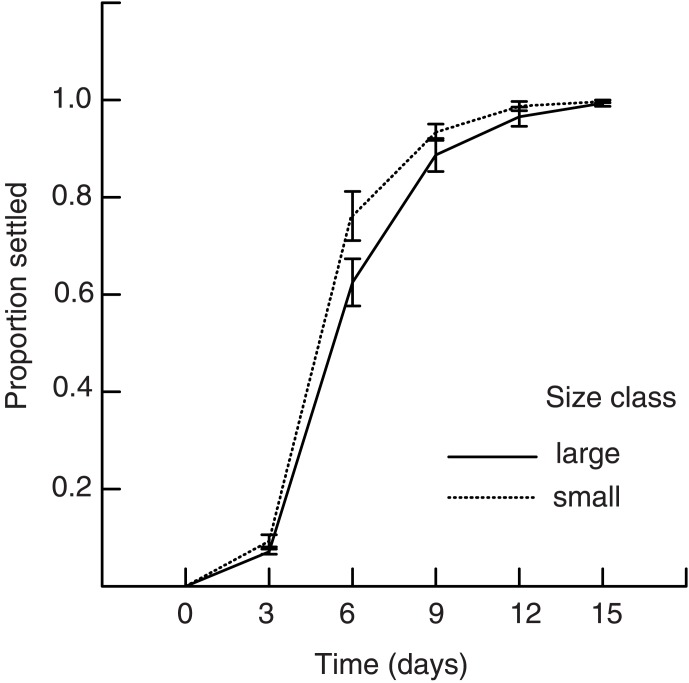
The relationship for larval settlement over fifteen days under conditions where *Hydroides diramphus* larvae were fed at concentrations of 100,000 cells ml^−1^ *Isocrysis*. Two treatments of larval size were applied: solid line represents the proportion of settlement of large larvae and the dashed line represents the proportion of settlement of small larvae. Error bars are ± SE.

Larval settlement increased over time (Figure 3, Table 1). Initially, settlement was slow with about 10% of larvae settling after three days. Average settlement increased to 70% between days three and six, and by day twelve almost all larvae had settled. The rate of settlement was also significantly affected by larval size, where larvae from smaller eggs settled sooner, and this effect was consistent across all time periods (no interaction between larval size and time on settlement; Table 1).

**Table 1:** Repeated measures analysis testing the effect of larval size on the proportion of *Hydroides diramphus* settlement over time.

Source	df	MS	*F*	*P*
Between Jars				
Larval size	1.000	0.006	5.523	0.030
Error	18.000	0.001		
Within Jars (across time)				
Time	4.000	0.274	413.555	<0.001†
Time × larval size	4.000	0.002	2.403	0.114†
Error	72.000	0.001		

† Greenhouse-Geisser adjusted *P*-values: ε = 0.429.

Larvae were fed a constant food source of *Isocrysis.*

### Egg size effects during post-metamorphosis

**Figure 4:**
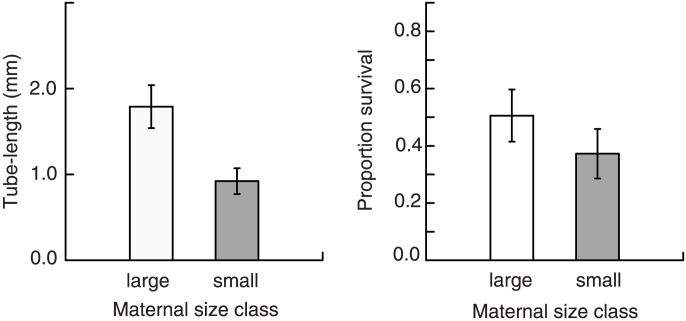
The effect of maternal size class on post-performance after 10 days under field conditions, for the tube worm *Hydroides diramphus*. (A) Post-metamorphic length of worms. (B) Post-metamorphic survival. Data presented untransformed. Error bars are ± SE.

After 10 days under field conditions, juveniles in the large size class (coming from large eggs) were on average longer in length than juveniles from the small size class (‘large’ 1.79 mm±0.23 SE and ‘small’ 0.92 mm±0.14 SE; *F*_1,14_ = 11.569, *P* = 0.004; Figure 4a). Overall survival of post-metamorphic juveniles was 44% after 10 days in the field, but there was no effect of size class on survival (*F*_1, 14_ = 1.411, *P* = 0.255; Figure 4b).

## Discussion

The results show *Hydroides diramphus* egg size effects can endure post-settlement and influence performance in early and late life-history stages. But, the effects of egg size on performance were variable both within and among life-history stages. On average, larger eggs had higher fertilization under sperm-limited conditions, were slightly faster to develop prior to feeding, and were larger post-metamorphosis. Considering these positive egg size-dependent performances, it might be expected that larger egg sizes should be favoured. However, smaller eggs had higher fertilization success when sperm was abundant, and a shorter planktonic development period overall, but egg size did not affect post-metamorphic survival. These negative and neutral effects of increased egg size suggest that in some conditions smaller egg sizes should be favored. Further, in a previous study on *H. diramphus*, egg size has been shown to interact in a highly complex and context-dependent manner with larval nutritional and post-metamorphic density [35]. Hence, egg size effects in *H. diramphus* demonstrate considerable conflicting performance effects depending on the environment and life-history stage that makes an optimal egg size difficult to predict.

High variability in offspring size effects are reported generally (see reviews: [3], [6]), and more specifically in marine invertebrates with similar performance proxies as *H. diramphus* [28], [29], [66]; and reviewed by [4], [60]. During fertilization in broadcast-spawners, egg size had been repeatedly shown to be under strong selection depending on local sperm concentrations [27], [43], [44]. Typically, larger eggs have higher fertilization success in sperm limited environments [4], and the results of the current study confirm this trend.

Development through sensitive embryonic and larval stages is also considered a major source of fitness variation in marine invertebrates, where longer developments times are thought to increase the probability of mortality [8]. Generally, larger embryos and smaller larvae that feed spend longer developing, although this trend is not ubiquitous [4]. The results of the present study demonstrate a negative relationship between egg size and egg cleavage rates, but differ from the general trend for the larval period, as larvae from smaller eggs were faster to settle. Classic life-history theory for marine invertebrates suggest that the relationship between the pre-feeding and feeding development periods should be inversely related [8], but this rests on the assumption that larvae settle at a similar sizes and differences in egg size do not persist post-metamorphosis. The results show that initial egg size was positively related to post-metamorphic size; hence the results for each developmental period and post-metamorphic size suggest the relationship among these traits and offspring size may depend on complicated tradeoffs. Two independent studies on egg size effects in the sea urchin *Strongylocentrous droebachiensis* showed evidence that such a tradeoff may exist. Sinervo and McEdward [36] found larvae from small eggs had longer planktonic periods, and metamorphosed at the same size as larvae from large eggs. But, Hart [67] found no effect of egg size on planktonic period, yet did show a small positive effect of egg size on size at metamorphosis. Explanations for why the results among two different studies on the same species are inconsistent are not known, but may be due to some other mediating environmental factor. Within a single experiment, *H. diramphus* has demonstrated that egg size effects are not straightforward and depended on complex interactions among the larval nutrition and post-metamorphic environments [35]. Other studies have shown that smaller offspring may use compensatory growth mechanisms to achieve comparable sizes at metamorphosis or maturity as larger offspring by prolonging development or accelerating growth/feeding rates [3], [17], [37], [38], [39]. Overall the dynamic and interdependent relationships among egg size and subsequent performance at later stages, demonstrate the need to assess the benefits of egg size and when egg size benefits are conflicting under the context of variable environments across the entire life history of the study organism.

## Supporting Information

File S1 (XLS)
